# Supplementary material for: Immunity to Sda1 Protects against Infection by Sda1+ and Sda1− Serotypes of Group A Streptococcus
Source: Vaccines (Basel). 2022 Jan 11;10(1):102. doi: 10.3390/vaccines10010102 (PMC8779841; doi:10.3390/vaccines10010102)
Supplement: Supplementary file 1 [file vaccines-10-00102-s001.zip › Supplementary_Figure_S3.pdf]

A

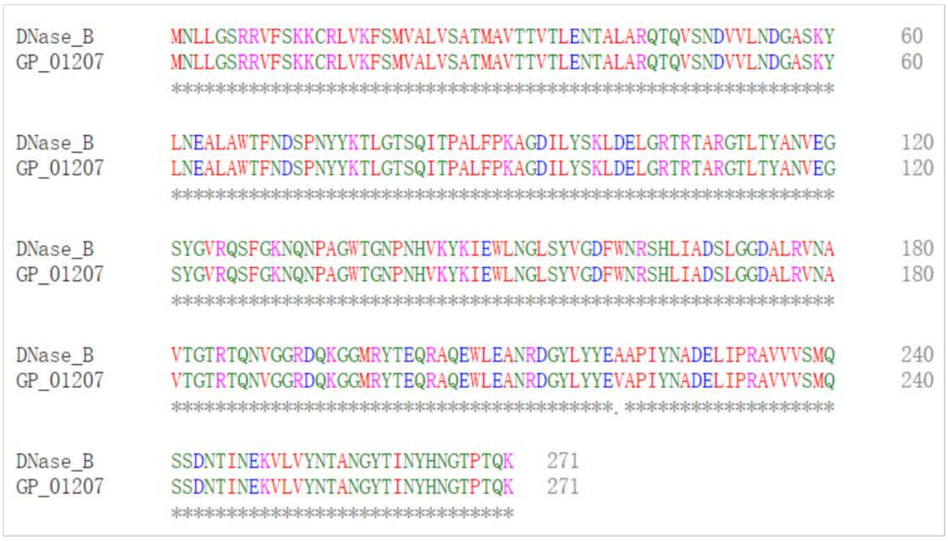

B

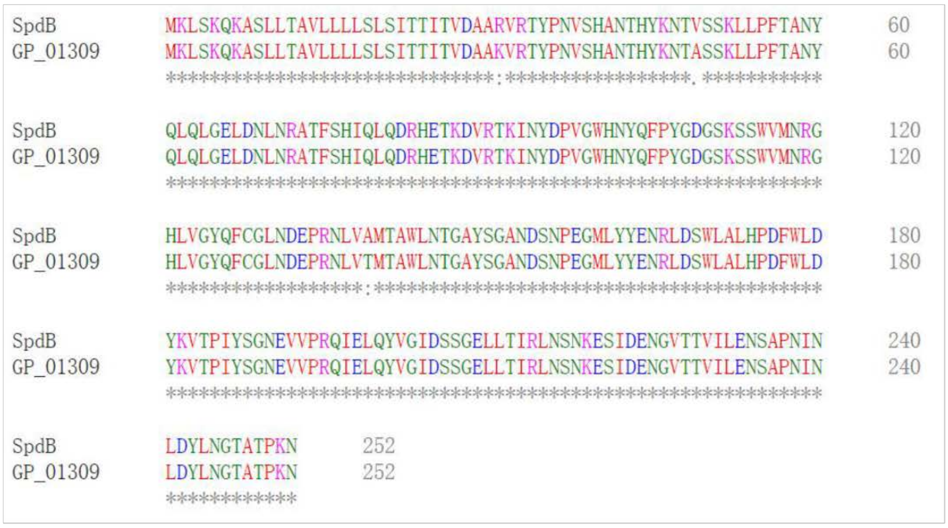

Figure S3. Alignments of DNase B (SpeF) and SpdB with amino acid sequences in proteome encoded by M3.11 genomic DNA. Alignment of amino acid sequence of DNase B (NCBI Reference Sequence: WP_010922721.1) with GP_01207 (amino acid sequence from GAS M3.11 proteome) A); and SpdB (NCBI Reference Sequence: WP_002985324.1) with GP_01309 (amino acid sequence from GAS M3.11 proteome) (B) by Clustal Omega (https://www.ebi.ac.uk/Tools/msa/clustalo/). Amino acid color

code obtained from Clustal Omega is as follows: red, small; blue, acidic; magenta, basic; green, hydroxyl or sulfhydryl or amine.
